# Isokinetic Strength Responses to Season-long Training and Competition in Turkish Elite Soccer Players

**DOI:** 10.2478/v10078-012-0017-5

**Published:** 2012-04-03

**Authors:** Niyazi Eniseler, Çağatay Şahan, Hikmet Vurgun, Hasan Fehmi Mavi

**Affiliations:** 1 School of Physical Education and Sports, Celal Bayar University, Manisa, Turkey.; 2 Department of Kinesiology and Sports Studies, Eastern Illinois University, Charleston, IL, USA.

**Keywords:** isokinetic strength, H/Q ratio, bilateral strength, soccer, seasonal changes

## Abstract

There are not enough studies that describe the isokinetic strength of professional soccer players at high angular velocities. The purpose of this study was to evaluate the seasonal changes in isokinetic strength of Turkish professional soccer players (n=14) over the course of a 24-week soccer season. The isokinetic strength of players who underwent usual soccer training and weekly competition throughout the soccer season was assessed by means of the Biodex System 3 dynamometer with the knee attachment. The peak torque of knee extensor and flexor muscles were measured at angular velocities of 60°/s, 300°/s and 500°/s. Players were tested at the beginning and end of the competitive season. While the first- and second-test measurements did not show significant changes at 60°/s and 300°/s angular velocities, at the end of the training period, players’ knee strength changed significantly at 500°/s angular velocities. In addition, the H/Q ratio improved significantly for the dominant as well as non-dominant leg at 500°/s. Significant bilateral strength improvements for knee flexors were also observed at 500°/s. The findings of this study suggest that usual daily soccer training (technical, tactical, power, strength, endurance, flexibility, etc.) and weekly competition might produce changes in knee strength at high angular velocities.

## Introduction

Soccer competitions demand activities such as kicking, jumping, heading, sprinting, turning, changing pace and tackling where players use explosive strength (Reilly and Thomas, 1976). In addition, the intensity of soccer competitions requires forceful contractions to keep balance and control of the ball against defensive pressure. All these activities need certain levels of strength and power from players in specific playing positions (Taskin, 2008). In fact, when observing soccer players during competitions who perform strength related activities, it may be noticed that they apply strength in varying high speeds (O’Donoghue, 2001). The strength of hamstring and quadriceps muscles are vital for soccer players for locomotion in the game. Previous research has consistently reported that concentric and eccentric strength of hamstring and quadriceps muscles may positively affect sprint and jumping performances of soccer players (Mark et al., 2004). Prior studies have also revealed that apart from skill, kicking performance in soccer was an important result of knee extension and flexion strength, as well as lower limb velocity (De Proft et al., 1988; Reilly and Drust, 1997). In addition, player injuries might result from the lack of strength of hamstring and quadriceps muscles and/or bilateral hamstring strength deficits combined with higher physical demans of competition and more aggressive play (Jönhagen et al., 1994). Therefore, attempts to train weak hamstring and quadriceps muscles to improve strength and achieve strength balance between them appear to be an important objective for coaches. In fact, players who are exposed to strength training can improve their muscular strength. For example, several researchers have found that technical and tactical training, games, conditioning and all other activities, combined with strength training, were shown to affect the strength levels of soccer players (De Proft et al., 1988; İşleğen and Akgün, 1988; Öberg et al., 1985; Reilly and Thomas, 1977). As a result of such a training program, strength gains among soccer players can be observed. For example, it was reported that regular players when compared to substitutes, and professional players when compared to amateurs, have superior isokinetic muscle strength at various angular velocities (Cometti et al., 2001; Wisloff et al., 1998). There are various studies in which researchers examined the pre-season physiological and physical changes of soccer players over a period of 6–8 weeks. Most of this research focused on endurance, power, and anthropometric measurements (Bangsbo, 1994; Brady et al., 1997; Brewer, 1990; Casajũs, 2001; De Proft et al., 1988) and limited research reported isokinetic strength changes at slower velocities in soccer players (Reilly and Thomas, 1977). However, no research examined the isokinetic strength changes at higher angular velocities in elite soccer players over a competitive season. Various environmental stimuli during soccer training and competition might result in certain long-term physiological adaptations to improve players’ endurance and speed, as well as strength. The biggest argument for including high-velocity exercise in a player’s resistance-training program deals with the concept of specificity (Murray, 2006). Soccer players apply high-speed movement for sprint performance during a match. Training with high-speed movement in strength exercises enables high-speed adaptation. Consequently, soccer players should practice their exercises at high speed. Also, high-velocity exercises may have an appropriate place in a periodized resistance-training program designed for players who require speed (Kawamori and Newton, 2006).

Therefore, the aim of this study was to evaluate the seasonal changes in isokinetic strength of knee muscles at various angular velocities in Turkish elite soccer players over the course of a 24-week soccer season that included regular daily practice and one or two regular matches per week. The authors attempted to answer the question how elite soccer players adapted to the effects of conditioning, practice, and high level competition over an entire season by assessing their strengths at two different time points.

## Material and methods

### Subjects

Fourteen soccer players, 18–32 years of age, all members of a professional soccer team in the Turkish Soccer Super League, participated in the study. Before conducting the experiment, all subjects were informed on the risks of the study and gave informed consent, and it was a part of their professional contract. The study was approved by the Ethics Committee of the Celal Bayar University and met the conditions of the Helsinki Declaration.

All soccer players participated in the 24-week soccer-specific training and competitions as regular and/or substitute players. Players with a long history of injuries were eliminated from the study. The basic physical characteristics of the players are summarized in Table 1.

### Procedures

Participants in this study were assessed at two different times throughout the 24-week soccer season. The first measurement of isokinetic strength was taken in the first week of soccer training (the first week of July). The second measurement was completed in the last week of the season (the last week of December). The Biodex System 3 (Biodex Medical Systems Inc., New York, USA) dynamometer was used to measure the isokinetic knee strength. Before testing, the participants were informed about the machine’s specific details and they warmed up for 15 minutes. Participants sat in an upright position on the adjustable dynamometer chair and the shoulders, pelvis and thighs of each subject were secured by straps to minimize extraneous body movements. Before testing each participant, the knee joint was positioned at 90^º^ of flexion (full extension defined as 0^º^) and the visual alignment of the dynamometer lever arm and the distal point of the lateral femoral condyle were completed. The movements were corrected by taking the effects of gravity into consideration. Participants held their arms in a comfortable position during testing. The measurement of peak isokinetic concentric knee extension and knee flexion torque in both legs were taken at 3 angular velocities of movement, low at 60°/s, intermediate at 300°/s, and high at 500°/s. The range of the knee extension and flexion contractions were performed at 0–90^º^. Testing progressed from slowest to highest speeds. Participants were given three opportunities to warm up at submaximal concentric contractions and then performed the maximal concentric contractions four times. They rested for 40 seconds before each angular velocity test.

The rest period between each lower limb testing lasted 3 minutes. Each participant was verbally encouraged during the test. Variables such as peak torque (Nm), knee flexor/knee extensor ratio (Hamstring/Quadriceps ratio (H/Q ratio)), bilateral leg strength differences as a percent of extensor (BDE) and flexor (BDF) were also calculated for further analysis. Bilateral leg strength differences were calculated by applying the Biodex System. The H/Q ratio was calculated by using the absolute values of each muscle as percentages. The highest peak torque was used to calculate the bilateral leg strength differences and H/Q ratio.

#### Soccer-Specific Training Content

The specific soccer training took 24 weeks to be completed, including 7 weeks of pre-season training and 17 weeks of in-season training during the second half of the soccer season. Table 2 and 3 summarize the season-long soccer-specific training content and general activity content of weekly training.

In general, as shown in Tables 2 and 3, typical soccer training included both general soccer training activities (technical and tactical training, games and matches) and conditioning (speed, agility, coordination, stretching, endurance and power development) during the pre-season as well as in season. The duration of practice sessions was 90–100 min when there was one training per day, while it was 60–70 min when there were two training sessions per day.

### Statistical Analysis

The mean ± standard deviation values were reported for all the variables used. A paired samples *t*-test was used to compare the differences between the first and second measures in this study. The level of significance was set at p<0.05.

## Results

### 

In terms of the physical characteristics of the participants (Table 1), no significant changes were observed for body mass between the first and second test measures for seasonal changes in peak isokinetic knee strength values of extensor and flexor muscles.

The first and second test values of the peak isokinetic strength, at 60°/s and 300°/s for knee extension and knee flexion, remained unchanged.

However, the peak isokinetic leg strengths at 500°/s for knee extension and knee flexion significantly increased (p<0.01) from the first to second evaluations (Tables 4 and 5).

#### Seasonal H/Q ratio changes

The comparisons of both dominant (D) and non-dominant (ND) legs hamstring to quadriceps ratio (H/Q ratio) mean values at 60°/s and 300°/s during the soccer season showed no significant changes (Table 6). However, an interesting finding was that a significant increase in the H/Q ratio at an angular velocity of 500°/s for both the D and ND legs (p<0.01) and of 300°/s for the D leg (p<0.05) were observed during the soccer season (Table 5). The H/Q ratio changed from 63.53±18.23% to 98.14±19.84% (p<0.01) for the dominant leg and increased from 72.97±15.59% to 96.36±19.15% (p<0.01) for the non-dominant leg at the 500°/s measurement level.

#### Seasonal Bilateral Strength Differences

No significant differences were observed at 60°/s and 300°/s for knee extension and knee flexion when comparing the average bilateral strength difference. However, the average bilateral strength differences at 500°/s for knee extension and knee flexion decreased (p<0.01) (Table 7). An examination of Table 6 at 500°/s angular velocities for pre-and post-season measurements illustrates that bilateral strength differences for knee extensors changed insignificantly from 14.86±7.58% to 11.37±9.09%. On the other hand, knee flexor bilateral strength differences changed significantly from 20.73±12.71% to 7.70±6.83% (p<0.01). Reported bilateral strength differences for knee extensor and flexor were also not significant at 60°/s as well as at 300°/s angular velocities.

## Discussion

The main finding of this study is that isokinetic strength performance increased at the end of the soccer season, as was shown by significant increases in isokinetic strength values at high angular velocities.

Observation of the soccer game shows that all important activities in soccer seem to be performed at high velocities. In fact, soccer players perform sprints every 90 seconds that can last 2–4 seconds and comprise up to 11% of the distance covered during a match (Reilly and Thomas, 1976; O’Donoghue, 2001). Knee movement reaches 600–700°/s (Sale, 1990) during high intensity efforts, such as sprinting. Soccer players also perform approximately 50 quick and forceful turns to maintain balance and control the ball under defensive pressure. Therefore, coaches probably consider the high velocity nature of soccer when constructing training programs to improve explosive strength of players. Indeed, velocity-specific training during strength training studies suggests that the training velocity should mimic athletic performance (Prevost et al., 1999). The effects of soccer-specific training on isokinetic strength at low angular velocities have previously been reported. However, no data exists that investigates isokinetic strength at high angular velocities over the course of a competitive season.

### Seasonal changes in peak isokinetic knee strength values for extensor and flexor muscles

The results of this study indicated that soccer-specific training activities and competitions did not change the peak knee isokinetic strength of the players at lower angular velocities. However, peak knee isokinetic strength changes in extensor and flexor muscles were reported at a 500°/s angular velocity (Tables 5 and 6), which was close to the velocities performed during training. Limited research shows that pre-season strength training might produce back and leg strength development (İşleğen and Akgün, 1988 ). In addition, some researchers reported the effects of 6 weeks pre-season strength training to increase knee and hip eccentric and concentric strength values at 3.65 rad·s^−1^ angular velocities as a result of strength training twice a week (1RM 80%) in addition to the normal soccer training throughout the season. Players in the strength training group showed an increase of 25% and 77% in eccentric and concentric strength while the control group players had an increase of 2% and 10% in their strength values (De Proft et al., 1988). Hoff and Helgerud (2004) also reported that a combination of soccer training and strength training (3 times a week, 85% of 1RM) led to a 52% increase in power development for soccer players without using the isokinetic dynamometer in strength measurements (Hoff and Helgerud, 2004).

Additionally, in the Hoff and Helgerud study (2004), the results of the players’ squat improved from 116 to 176 kg and their vertical jumping height increased from 57.2 to 60.2 cm (Hoff and Helgerud, 2004). Although there are reports that pre-season and in-season strength training combined with specialized soccer training might increase the strength of lower extremities in players, there are also studies with controversial findings. For example, Reilly and Thomas (1977) reported that 6 weeks of pre-season training did not contribute to strength improvement (Reilly and Thomas, 1977). Although our study differs from the studies mentioned above in terms of methods, the way the strength was measured (isokinetically) and measurement velocities (60°/s, 300°/s and 500°/s), strength changes were observed as a result of the daily regular soccer training program and weekly competitions. Our results also suggest that soccer-specific training and competitions might be important for producing knee isokinetic strength changes at high angular velocity (at 500°/s). Nevertheless, velocity specificity of resistance training is controversial, and training with high velocity movements increases high-velocity strength relatively more than low-velocity strength, and vice versa (Kanehisa and Miyashita, 1983; Narici et al., 1989; Sale, 1992). In contrast, other studies have shown that high-velocity training also produces significant increases at low angular velocities (Dudley and Djamil, 1985). In fact, velocity specificity of resistance training has demonstrated that the greatest strength gains occur at or near the training velocity (Behm and Sale, 1993).

### Seasonal H/Q ratio changes

Maximal isokinetic hamstring muscle strength relative to the maximal isokinetic quadriceps muscle strength ratio (H/Q ratio) is used regularly to describe the muscle strength properties for the knee joint (Kannus, 1994). As this study points out, specific soccer training results in H/Q ratio changes in the dominant as well as non dominant leg at 500°/s angular velocities (Table 6). Reported differences were not significant at 60°/s as well as at 300°/s angular velocities, except for 300°/s in the dominant leg. As the results indicate, the H/Q ratio changes were close to the strength training velocity of each player. Previous studies claimed that leg flexion and leg extension strength levels should be equal to one another and the hamstring muscle group is the weak link in sprinting, therefore, it needs to be strengthened (Dintiman and Ward, 1988). In addition, a low H/Q torque ratio is one of the factors that might contribute to injuries (Jönhagen et al., 1994). As our study shows, a soccer-specific training program and competitions produce a change in H/Q torque ratio at higher angular velocities. Strengthening the hamstring/quadriceps muscle group to achieve a 1:1 torque ratio could be important for muscular stability at the knee joint during fast knee extension (Aagaard et al., 1995). Metaxas et al. (2009) found that quadriceps and hamstring peak torques at various angular velocities of 60, 180, and 300°/s were 270.4±31.4 (Nm), 178.9±21.1(Nm), 128.6±14.5 (Nm) for quadriceps and 155.2±13.9 (Nm), 104.2±13.2 (Nm), 77.2±14.7 (Nm) for preparatory period in 100 soccer male players from the Greek national leagues.

### Seasonal Bilateral Strength Changes

In this study, the results show that soccer-specific training and weekly competitions were effective in reducing the imbalance between the dominant and non-dominant legs, especially at 500°/s angular velocities. This could be beneficial to soccer players in improving their performance and, most importantly, reducing the risk of injury in their lower limbs. As indicated, differences in strength profiles between the two legs have been considered as an important predictor of injury in soccer players (Leatt et al., 1987).

In fact, a bilateral imbalance of hamstring and quadriceps muscle strength between the dominant and non-dominant legs has been found to be at the level of 10% in soccer players (Hamzeh and Head, 2004). A muscle strength imbalance of more than 10% might increase the risk of injury (Kramer and Balsor, 1990). In our study, as shown in Table 7, while bilateral strength differences for the knee extensor was 11.37±9.09%, knee flexor bilateral strength differences were less than 10% (7.70±6.83%) at 500°/s for post-season measurement.

### Possible reasons for isokinetic knee strength changes

In summary, in the present study, there was a statistically significant change in peak knee isokinetic strength levels and H/Q ratios only for flexors at 500°/s angular velocities (especially for knee flexors). On the other hand, bilateral strength differences decreased significantly. The reasons for finding changes at high angular velocities include (1) performing all strength training at higher velocities at least once per week, (2) performing extra strength training for hamstrings (which was the weak knee flexor muscle group) to achieve a higher H/Q ratio, (3) performing leg curls for hamstrings, and leg extensions for quadriceps by choosing weight intensities close to 1/1 to improve the H/Q strength ratio, and (4) performing high speed strength training (power training), sprint, agility, coordination, and plyometric exercises throughout the season, twice a week. Another potential explanation for the high change might be that players had very low pre-season strength levels. Previous studies claimed that explosive strength changes might be the result of learning (Rutherford and Jones, 1996), improved coordination (Sale, 1992), and a reduction in antagonist co-contraction (Wisloff et al., 1998). In addition, Poulson et al. (1999) reported that a drop in a co-activation level, which occurred close to the mean angular velocity of training, may partly explain the arm flexion strength gains. Changes in high angular velocity strength production during the 24-week soccer-specific training and weekly competitions might result primarily from significant increases in the neural adaptation of the trained muscles. Previous research findings supported the idea of the neural adaptation of muscles as a result of a specific training regimen. For example, Behm and Sale (1993) claimed that high velocity training adaptations might involve significant neural adaptations (Behm and Sale, 1993). In addition, Häkkinen et al. (1985) showed significant improvements in strength and power with light-load explosive resistance training (Häkkinen et al., 1985). Such developments were supported by observation of electromyography increases in muscle electrical activities primarily at the velocity of training. Specifically, it appears that the velocity of the movement, as controlled by the load, plays a key role in improving high-velocity performance capabilities and possible neural mechanisms of adaptation (Häkkinen et al., 1985). Prevost et al. (1999) also reported short-term improvements in isokinetic torque at high velocities following training that involved fast isokinetic contractions, which may occur due to neural facilitation. However, the reader should be aware of the arguments that neural adaptations may be limited to the initial stage of strength training (6–8 weeks) (Sale, 1992) or such adaptations may continue during the later stages of strength training (Hoff et al., 2001).

In conclusion, our data suggests that the 24-week planned program of soccer-specific conditioning and weekly competitions may help soccer players to have significant strength gains at high angular velocities of 500°/s. However, at lower angular velocities (60°/s and 300°/s), there were either no or very limited strength gains for knee extensor and flexor muscles.

Specifically, at a 500°/s angular velocity, while the peak isokinetic leg strength for knee extension and flexion and H/Q ratio for dominant and non-dominant legs were improving, bilateral strength differences were decreasing. Thus, the changes showed that daily soccer-specific training and weekly competitions might increase strength at high angular velocities.

## Figures and Tables

**Table 1:** Basic characteristics of the players (n=14).

	First test	Second test
Age (years)	25.8±3.9	26.3±3.9
Body Mass (kg)	75.1±3.3	75.5±4.3

Values are means (SD)

**Table 2:** Overview of season-long soccer specific training content

Type of activity	Percentage of each training component
Stretching	10.26
Strength training (power training)	8.27
Endurance training (aerobic and soccer specific endurance)	12.91
Speed, coordination (various shuffles) and agility	18.21
Plyometrics	7.66
Technical training	11.58
Tactical preparation	7.94
Games	23.17
Soccer-match	7.61

**Table 3:** General activity content of weekly training

**Weekly training content**	
**Monday**	Mainly rest (if not, aerobic training and flexibility, sauna and massage were the choice of activities)
**Tuesday**	Interval training or aerobic endurance, game Morning session: Power training.
**Wednesday**	Afternoon session: Circuit training that includes agility exercises and game
**Thursday**	Plyometrics or speed, tactical training and game
**Friday**	Technical and tactical training
**Saturday**	Stretching and technical training
**Sunday**	Competition

**Table 4:** Changes in angle-specific peak isokinetic knee strength of quadriceps for both D and ND legs over the 24-week soccer season

Extensor Peak torque (Nm)						
	60°/s		300°/s		500°/s	

	Dominant	Non-Dominant	Dominant	Non-Dominant	Dominant	Non-Dominant
Initial test	272.72±38.98	271.61±38.66	148.15±15.40	145.04±20.5	79.92±15.42	73.63±12.05
Final test	253.03±42.16	250.93±54.24	148.96±19.73	146.76±17.07	135.84±17.18	150.1±22.09
	p>0.05	p>0.05	p>0.05	p>0.05	p<0.01^**^	p<0.01^**^

n = 14; mean ± SD,

* p<0.05,

** p<0.01 significant differences between the initial and final test.

**Table 5:** Changes in angle-specific peak isokinetic knee strength of hamstring for both D and ND legs over the 24-week soccer season

Flexor Peak torque (Nm)						
	60°/s		300°/s		500°/s	

	Dominant	Non-Dominant	Dominant	Non-Dominant	Dominant	Non-Dominant
Initial test	150.49±19.92	148.24±15.30	94.83±12.37	96.78±14.70	50.96±16.34	51.72±14.33
Final test	177.97±64.84	158.86±40.93	96.83±14.17	107.15±12.38	133.6±20.96	140.1±21.83
	p>0.05	p>0.05	p>0.05	p>0.05	p<0.01^**^	p<0.01^**^

n = 14; mean ± SD,

* p<0.05,

** p<0.01 significant differences between the initial test and final test.

**Table 6:** Changes in angle-specific hamstring to quadriceps ratio (H/Q ratio) for both D and ND legs over the 24-week soccer season

H/Q ratio (%)						
	60°/s		300°/s		500°/s	

	Dominant	Non-Dominant	Dominant	Non-Dominant	Dominant	Non-Dominant
Initial test	55.40±7.80	55.54±6.32	64.1±6.98	67.45±10.84	63.53±18.23	72.97±15.59
Final test	62.73±23.68	60.81±19.13	75.19±11.65	74.05±12.62	98.14±19.84	96.36±19.15
	p>0.05	p>0.05	p<0.05^*^	p>0.05	p<0.01^**^	p<0.01^**^

n = 14; mean ± SD,

* p<0.05,

** p<0.01 significant differences between the initial test and final test.

**Table 7:** Changes in angle-specific bilateral leg strength differences of extensor and flexor over the 24-week soccer season

Bilateral strength differences ratio (%)						
	60°/s		300°/s		500°/s	

	Extensor	Flexor	Extensor	Flexor	Extensor	Flexor
Initial test	9.25±6.73	9.27±6.73	12.25±4.46	12.57±9.88	14.86±7.58	20.73±12.71
Final test	8.80±8.97	8.80±8.97	10.46±7.53	8.42±5.66	11.37±9.09	7.70±6.83
	p>0.05	p>0.05	p>0.05	p>0.05	p>0.05	p<0.01^**^

n = 14; mean ± SD,

* p<0.05,

** p<0.01 significant differences between the initial test and final test.
